# Barriers and facilitators to maintaining a high level of polypharmacy adherence in people living with HIV: A scoping review

**DOI:** 10.3389/fphar.2023.1013688

**Published:** 2023-03-02

**Authors:** Jiamin He, Zheng Zhu, Meiyan Sun, Xiaoning Liu, Junwen Yu, Lin Zhang, Hongzhou Lu

**Affiliations:** ^1^ School of Nursing, Fudan University, Shanghai, China; ^2^ Department of Nursing, Shanghai Public Health Clinical Center, Shanghai, China; ^3^ Fudan University Center for Evidence-based Nursing: A Joanna Briggs Institute Center of Excellence, Shanghai, China; ^4^ Rory Meyers College of Nursing, New York University, New York, NY, United States; ^5^ Department of Infectious Disease, National Clinical Research Center for Infectious Diseases, Shenzhen Third People’s Hospital, Shenzhen, Guangdong, China; ^6^ Faculty of Medicine, National Heart and Lung Institute, Imperial College London, London, United Kingdom

**Keywords:** HIV, aids, polypharmacy, adherence, scoping review

## Abstract

**Objectives:** With the prolongation of life span and increasing incidence of comorbidities, polypharmacy has become a challenge for people living with HIV/AIDS (PLWH). This review aimed to identify barriers and facilitators to maintaining a high level of polypharmacy adherence in people living with HIV/AIDS.

**Methods:** Nine electronic databases were searched for studies from 1996 to October 2021. Studies were included if they were conducted with adults living with HIV/AIDS and reported barriers and facilitators to maintaining a high level of polypharmacy adherence. This review presents a conceptual framework model to help understand the barriers and facilitators.

**Results:** Twenty-nine studies were included. The majority of publications were observational studies. Eighty specific factors were identified and further divided into five categories, including individual factors, treatment-related factors, condition-related factors, healthcare provider-related factors, and socioeconomic factors, based on the multidimensional adherence model (MAM).

**Conclusion:** Eighty factors associated with polypharmacy adherence among people living with HIV/AIDS were identified and grouped into five major categories. Healthcare providers can make decisions based on the five categories of relevant factors described in this paper when developing interventions to enhance polypharmacy adherence. It is recommended that medications be evaluated separately and that an overall medication evaluation be conducted at the same time to prevent inappropriate polypharmacy use.

## 1 Introduction

As a result of the widespread use of antiretroviral therapy (ART), the average life expectancy of people living with HIV/AIDS (PLWH) has been greatly extended (Ghosn et al., 2018). However, due to various reasons, such as weakened immunity and aging, PLWH often have concomitant diseases and symptoms (Zhu et al., 2019). PLWH need to take a variety of medications for other diseases and conditions, in addition to ART drugs, resulting in long-term use of multiple medications (Gallardo-Anciano et al., 2019).

Polypharmacy is becoming an increasingly concerning issue in PLWH, especially among older PLWH (Guaraldi et al., 2018). There is no standard definition for polypharmacy in PLWH. Danjuma et al. (2020) conducted a systematic review and found that there were 36 definitions for polypharmacy in PLWH, most of which defined polypharmacy as the concomitant use of five or more medications, and most of which excluded ART medications. David and colleagues defined polypharmacy as the use of non-HIV drugs in addition to antiretroviral drugs (Back and Marzolini, 2020). Although the definition of polypharmacy is not unified, it is clear that polypharmacy relates to both the types of drugs used and the number of tablets.

Polypharmacy not only results in a heavy pill burden for PLWH and increases the complexity of medication regimens but also increases the risk of drug–drug interactions and hospitalization (El Moussaoui et al., 2020). Studies have shown that complex treatment regimens (Manzano-García et al., 2018), a heavy pill burden (Yager et al., 2017), and medication side effects (Krentz and Gill, 2016) are associated with lower polypharmacy adherence in PLWH. In addition, PLWH with polypharmacy often have multiple comorbidities, which aggravate the patient’s condition and psychological burden, resulting in decreased polypharmacy adherence (Manzano-García et al., 2018; Gervasoni et al., 2019). Most previous studies have reported adherence to ART medications and non-ART medications separately in PLWH with polypharmacy. Only one study from the United States reported overall medication adherence, showing that only 50% of PLWH achieved 85% adherence to multiple different medications (Zelnick et al., 2021). Non-compliance with medication can prevent patients from achieving good outcomes and increase hospitalization and mortality rates (Altice et al., 2019).

Over the past three decades, most studies on medication adherence in PLWH have focused on ART medication use while ignoring concomitant non-ART medication use, which is increasingly important and prevalent in PLWH (Paramesha and Chacko, 2021). Some studies have explored barriers and facilitators to maintaining a high level of polypharmacy adherence, but these studies focused on different factors and reached different conclusions. This scoping review aimed to identify barriers and facilitators to maintaining a high level of polypharmacy adherence in PLWH and develop conceptual models of polypharmacy adherence. This may be useful for the clinical development of effective intervention strategies.

## 2 Methods

### 2.1 Data sources and search strategy

A comprehensive three-step search strategy was adopted to identify relevant literature. First, a limited search was conducted in the PubMed/Medline and WanFang databases (Chinese). Second, a comprehensive search was conducted using the following electronic databases and gray literature sources: PubMed/Medline, Embase (Ovid), CINAHL (EBSCO), Web of Science, Cochrane Library (Wiley), ProQuest Dissertations, WanFang database (Chinese), CNKI (Chinese), and SinoMed (Chinese). The search time frame was set from January 1996 to October 2021. The starting point was 1996 due to the timing of the wide application of ART in PLWH. The search strategy was tailored to the specific requirements of each database. The search strategy for all databases is available in Supplementary File S1. Finally, a manual search of the references of the included studies was performed.

### 2.2 Eligibility criteria

Studies were included if they 1) aimed to explore barriers and facilitators associated with polypharmacy adherence in HIV-infected people; 2) were conducted with adults living with HIV/AIDS aged over 18 years old; and 3) were published in English or Chinese. Quantitative, qualitative, and mixed-method studies were considered. Literature reviews and systematic reviews were excluded. Studies were excluded if they reported only comorbidities and not concomitant medications. Studies that included populations other than PLWH or populations with comorbid HIV infection were included if outcomes were reported separately for the subgroup with HIV infection. Studies that included populations under 18 years old were included if outcomes were reported for populations over 18 years old. We defined polypharmacy as the simultaneous use of two or more different types of medications (including or not including ART drugs) in line with the literature review results and medication characteristics of PLWH. Therefore, we further excluded studies that reported “multiple pill adherence” based only on the number of drugs, which was defined as the number of pills taken as prescribed, regardless of the types of medications.

### 2.3 Study selection

**FIGURE 1:**
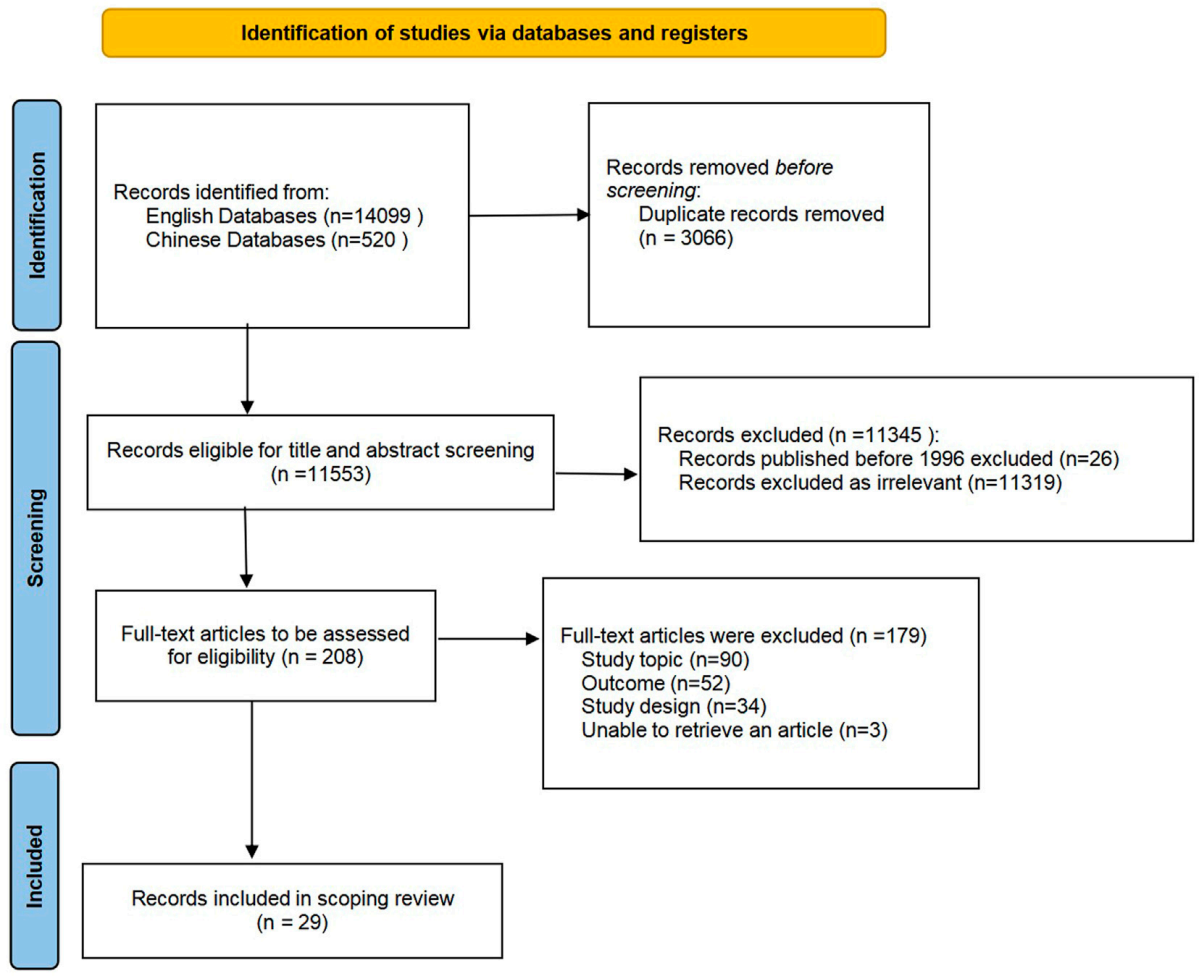
Flow diagram of study selection.

All citations were imported into Endnote X9 software (Thomson Corporation, United States), and duplicates were removed. A two-stage screening process was used to assess the relevance of studies identified in the search. First, two reviewers (JH and JY) independently reviewed the titles and abstracts according to the eligibility criteria. Second, the full texts of studies selected by both reviewers were procured for subsequent screening. Disagreements were resolved by discussion between reviewers or with the involvement of a third reviewer (ZZ). We used a Preferred Reporting Items for Systematic Reviews and Meta-Analyses extension for Scoping Reviews (PRISMA-ScR) flow diagram (Figure 1) to present the search process.

### 2.4 Data extraction

One reviewer (JH) extracted and summarized data from the included studies, and two reviewers checked the data for accuracy (XL and MS). The collected information included the following: author, publication year, sample size, county of origin, study design, age, sex, study settings, measures of medication adherence, the threshold for measurement, barriers and facilitators associated with polypharmacy adherence, and other key findings. In addition, information regarding the effectiveness of the interventions was extracted.

### 2.5 Data analysis

The descriptive statistics are reported in tabular form. The barriers and facilitators from the quantitative study results were labeled with effect sizes and 95% confidence intervals. Regarding factors from the qualitative study and the discussion and summary of the quantitative study, only the relevant factors are listed.

The barriers and facilitators identified in this scoping review were based on the multidimensional adherence model (MAM) organized by the World Health Organization (WHO). The MAM is used to examine treatment adherence from multiple and holistic perspectives and is the most appropriate model for determining medication adherence (Aldan et al., 2022). The MAM framework comprises individual factors, treatment-related factors, condition-related factors, healthcare provider-related factors, and socioeconomic factors (Goh et al., 2017). We also classified all factors into four categories (Ghosn et al., 2018): barriers to maintaining a high level of polypharmacy adherence (Zhu et al., 2019); facilitators to maintaining a high level of polypharmacy adherence (Gallardo-Anciano et al., 2019); inconsistent factors; and (Guaraldi et al., 2018) non-associated factors. Identified interventions were classified into the four categories described above depending on the outcome of their effect. Although some factors did not show a significant correlation in the studies related to polypharmacy compliance in PLWH, they may also be potential non-compliance correlation factors, and further studies may be needed to explore the correlation; therefore, factors with no significant association found in the literature were also included. Our scoping review was conducted following the JBI methodology for scoping reviews and the PRISMA-ScR guidelines (Peters et al., 2015; Tricco et al., 2018).

## 3 Results

### 3.1 Study identification

Figure 1 shows the study identification and selection process. A total of 14,619 records were retrieved from the electronic databases. After duplicates were removed, a total of 11,553 records remained for the screening of titles, abstracts, and full texts. Ultimately, 29 studies were deemed eligible and included in the scoping review.

### 3.2 Characteristics of the included studies

The characteristics of the 29 included studies are summarized in Table 1. There were 23 observational studies. The included studies were conducted in a wide range of locations, including five continents and nine countries. Most studies were conducted in North America (*n* = 13), followed by Europe (*n* = 7), Africa (*n* = 7), Oceania (*n* = 1), and Asia (*n* = 1).

**TABLE 1:** Summary of study results.

Study, year	Sample size	Location	Study design	Age (years)	Males, n (%)	Setting	Assessor	Adherence measurement threshold
Zelnick, 2021	198	United States	Cohort study	35 (29-43)^b^	85 (42.9)	Hospital	EDM	85%; Based on missed pill openings over past 6 months
Abdu, 2021	556	Ethiopia	Case–control study	41.64 (11.7)^a^	165 (39.6)	Hospital	Self-reports	95%; Based on the dose adherence scale by the Ethiopian Federal Ministry of Health
Morillo-Verdugo, 2021	61	Spain	Quasi-experimental study	53 (51-58)^b^	44 (72)	Hospital	SMAQ, e-ARMS, dispensation records	90%; Based on dispensing records during a six-month follow-up period and a positive questionnaire score
Gimeno-Gracia, 2020	74	Spain	Cross-sectional study	69 (66.7–72)^b^	64 (86.5)	Outpatient pharmacy clinic	SMAQ, MGQ, dispensation records	95% (for ART), >90% (for non-ART); Based on possession rate over the last 6 months
Khawcharoenporn, 2020	248	Thailand	Cohort study	37 (28-47)^a^	177 (71)	Hospital	Pill counts and 3-item CASE	100%
Saravolatz, 2019	163	United States	Case–control study	36.5 (10.8)^a^	119 (73)	Hospital	Laboratory values	Defined as having neither immunological failure nor virological failure
Manzano-García, 2018	619	Spain	Cross-sectional study	48 (43-45)^b^	504 (81.4)	Hospital	SMAQ, MMAS, dispensation records	95%; Based on dispensing records and an MMAS score of 4
Siefried, 2018	522	Australia	Cross-sectional study	50.8 (12.3)^a^	494 (94.6)	Sexual health clinics, hospital clinics, and general practice sites	Self-reports	100%; Based on patient-reported interruptions in the previous 12 months
Monroe, 2018	46	United States	Randomized controlled trial	Control group: 52.6 (0.9)^a^	Control group: 13 (57%)	HIV clinic	Patient self-reports (baseline) and dispensing records	80%; Based on the MPR for the 6-month interval before and after the intervention
				Intervention group: 51.96 (5.2)^a^	Intervention group: 14 (61%)			
Borrego, 2018	598 (333)	Spain	Cohort study	48.8 (8.8)^a^	262 (78.9)	Hospital	Dispensing records	90%; Based on PDC in the last 3 months
Kamal, 2017	105	Switzerland	Cross-sectional study	56 (51-63)^b^	74 (70)	Hospital	Self-reports, SHCS-AQ	100%; Not missing any dose in the past 4 weeks
Yager, 2017	1,202 (STR: 165; MTR: 1,037)	United States	Cohort study	50.6 (8.9)^a^	1,168 (97.2)	VISN-2	Electronic pharmacy refill records	95%; Based on dividing the number of adherent days by the total length of the regimen/prescription (days)
Krentz, 2016	1,329	Canada	Cohort study	(>16)^c^	1,008 (75.8)	Clinic	Dispensing records	100%; Based on pharmacy refill data
				≤30: 8.1%				
				31-40: 25.7%				
				41-50: 37.1%				
				51-60: 21.7%				
				>60: 7.4%				
Jiménez Galán, 2016	89	Spain	Cross-sectional study	50.04 (45.79–56.41)^b^	68 (76.4)	Outpatient pharmacy care unit	Dispensing records	90%; (adherence to ART)
								80%; (adherence to lipid lowering treatment)
Casaletto, 2016	50	United States	Cohort study	47.1 (9.7)^a^	44 (88)	Hospital	MEMS	90%; Based on bottle openings
Ayele, 2016	162	Ethiopia	Cohort study	36 (NA)^b^	74 (45.7)	Hospital	Pill count and diaries	90%; Based on pills taken during the 6-month course of treatment
Kalichman, 2015	123 CESD Score <16 (N = 71); CESD Score >16 (N = 52)	United States	Cohort study	46.9 (8.7)^a^	78 (63)	Community services and infectious disease clinics	Pill count	Based on unannounced pill counts over the 6-week observation period
Calvo-Cidoncha, 2015	66	United States	Cohort study	47 (4.8)^a^	57 (86.4)	2 Hospitals	Electronic pharmacy refill records	95%; Adherence before the addition of anti-HCV therapy to HAART was assessed for 6 months. After the introduction of anti-HCV therapy into HAART, adherence was assessed from the initial prescription of anti-HCV therapy until its date of discontinuation
Cantudo-Cuenca, 2014	594	Spain	Cohort study	47 (43-51)^b^	476 (80.1)	Hospital	Dispensing records and the MMAS	90%; Based on dispensing records and an MMAS score of 4
O’Donnell, 2014	104	South Africa	Cohort study	35 (18-60)^e^	50 (48)	Specialist hospital	Self-reports	100%; Based on the previous 7 days
Mangesho, 2014	124	Tanzania	Qualitative study	NA	38 (31)	Hospital	Self-reports	NA
Daftary, 2014	23	South Africa	Qualitative study	36 (NA)^b^ (18-56)^e^	10 (43)	Hospital	Self-reports	NA
Kebede, 2012	24	Ethiopia	Cross-sectional study	32.4 (9.6)^a^	11 (45.8)	Hospital	Self-administered questionnaires	Based on the frequency of missed doses per month (≤3 times)
Yi, 2011	1,094	United States	Cross-sectional study	≤30: 4.2%	0	HIV care and testing sites, drug and TB treatment programs, community-based organizations, sexually transmitted disease clinic programs	Self-reports	100%; Based on patients’ reports of nonuse
				31-40: 25.7%				
				41-50: 43.9%				
				>50: 26.2%^d^				
Gebremariam, 2010	24 (15 patients)	Ethiopia	Qualitative study	NA	8 (53)	Health Center	Self-reports	NA
Kumar, 2009	1,192	United States	Cohort study	(18-50+)^c^	898 (80.60)	Agency or clinic	Self-reports	100%, 85%, 57%; Based on the highest number of days on which doses were missed over the past 7 days
				18-34: 32.0%				
				35-50: 56.1%				
				>50: 11.9%				
Walkup, 2007	406	United States	Cohort study	(30-39)^c^	214 (52.4)	AIDS/HIV Registry	Medical records	100%; Based on a person-month approach
Cohn, 2002	643	United States	Randomized controlled trial (RCT)	40 (NA)^b^	561 (87)	University-based clinical centers	Self-reports	① “Non-adherence to MAC prophylactic study medication” was defined as missing ≥1 dose during the preceding 2 weeks
								② “Non-adherence to antiretroviral therapy” was defined as missing any doses of antiretroviral medication during the prior 48 h
Eldred, 1998	244	United States	Cross-sectional study	(≥18)^c^	154 (63.1)	HIV hospital-based clinic	Medical records and Patient self-reports	80%; Self-report of taking ≥80% of the prescribed therapy in the past week or self-reports of taking the prescribed therapy for ≥80% of days in the past 2 weeks

Abbreviations: ART, antiretroviral therapy; AIDS, acquired immunodeficiency syndrome; CASE, center for adherence support evaluation; CESD, centers for epidemiological studies depression scale; e-ARMS, electronic Adherence to Refills and Medications Scale; EDM, electronic dose monitoring; HAART, highly active antiretroviral therapy; HCV, hepatitis C virus; HIV, human immunodeficiency virus; MGQ, Morisky-Green questionnaire; MMAS, morisky medication adherence scale; MPR, medication possession ratio; MEMS, medication event monitoring system; MAC, *mycobacterium avium* complex; MTR, multiple tablet regimen; NA, data not available; PDC, proportion of days covered by medication; SMAQ, simplified medication adherence questionnaire; SHCS-AQ, Swiss HIV cohort study adherence questionnaire; STR, single-tablet regimen; TB, tuberculosis; VISN-2, Upstate New York Veterans’ Healthcare Administration; a, expressed as the mean (SD); b, median age years (IQR, interquartile range); c, age range; d, *n* (%); e, median age years (range).

The twenty-nine included studies enrolled a total of 10,744 participants (6,932 male and 3,812 female participants). The average sample size was 370, ranging from 23 to 1,329 (Daftary et al., 2014; Krentz and Gill, 2016). Of the 22 studies that reported the mean or median ages of the included population, only one included people with an average age over 65 years (Gimeno-Gracia and Sanchez-Rubio-Ferrandez, 2020), and the rest included people with median ages between 32.4 and 56 years (Kebede and Wabe, 2012; Kamal et al., 2018). Most studies were conducted in hospitals (*n* = 17), followed by HIV or infectious disease clinics (*n* = 4), agencies or communities (*n* = 3), multiple settings (*n* = 3), and outpatient pharmacy clinics (*n* = 2).

Polypharmacy adherence was measured by participant self-reports (*n* = 19), medication possession ratios (MPRs) calculated using electronic medical records or pharmacy dispensing records (*n* = 12), pill counts (*n* = 3), electronic monitoring using medication event monitoring systems (MEMS) (*n* = 2) and laboratory values (*n* = 1), as detailed in Table 1. Participants’ self-reported measurements of compliance referred to the use of questionnaire measurements, including the Simplified Medication Adherence Questionnaire (SMAQ), the Morisky-Green questionnaire/Morisky Medication Adherence Scale (MMAS), a self-developed questionnaire, and diaries (Ayele et al., 2016). Three qualitative studies obtained compliance outcomes through focus group interviews and in-depth individual interviews (Gebremariam et al., 2010; Daftary et al., 2014; Mangesho et al., 2014).

### 3.3 Barriers and facilitators

**FIGURE 2:**
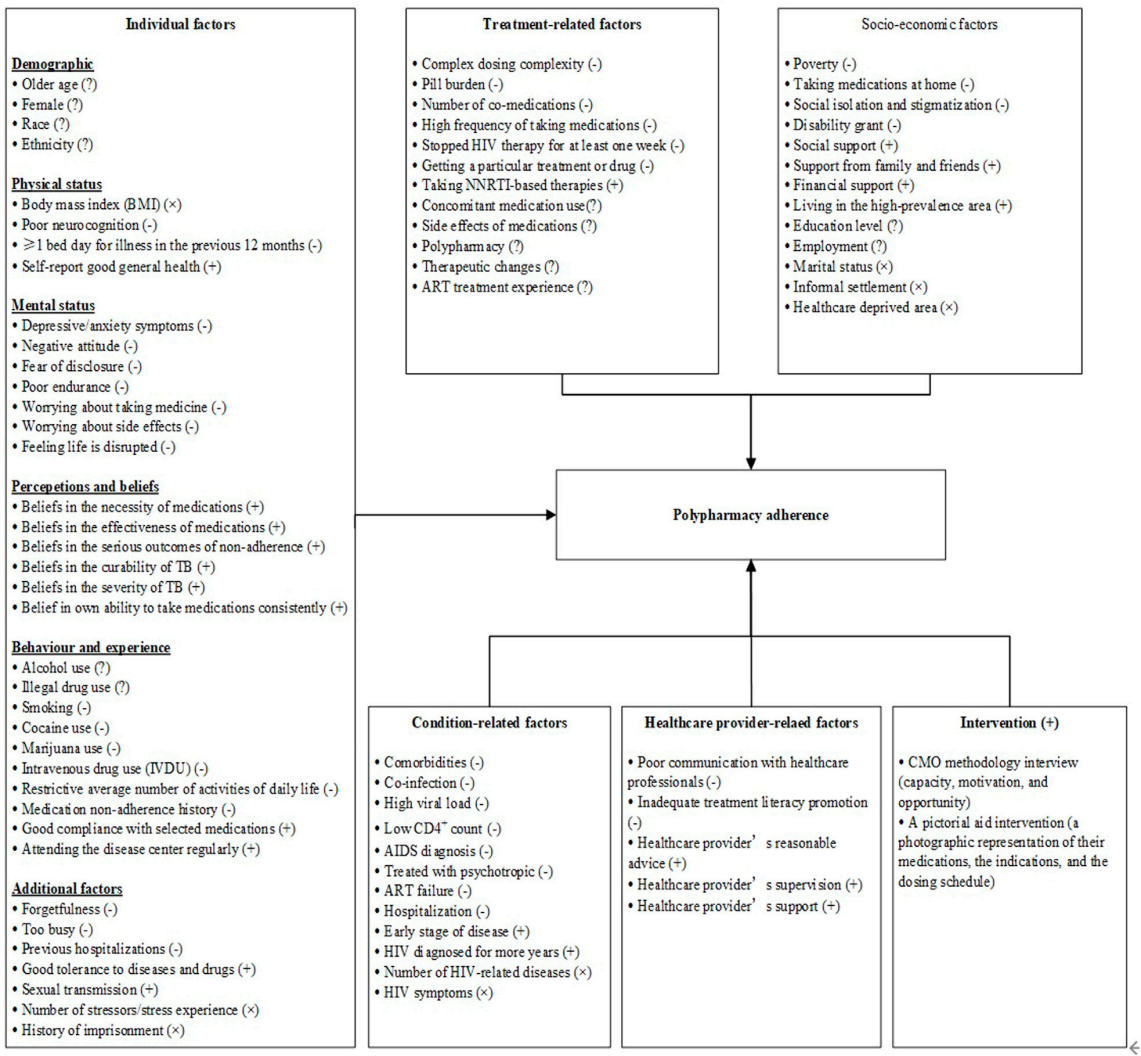
A multidimensional model for barriers and facilitators to maintaining a high level of polypharmacy adherence.

A total of 80 reviewed factors (see Supplementary Table S1) were grouped into five categories: individual factors, condition-related factors, treatment-related factors, health provider-related factors, and socioeconomic factors. Furthermore, we divided factors of compliance with polypharmacy adherence in PLWH into four categories, namely, barriers (−), facilitators (+), inconsistent factors (?), and not significantly associated factors (×), as detailed in Figure 2.

#### 3.3.1 Individual factors

Individual factors were the most important factors affecting polypharmacy adherence in PLWH, as most barriers, facilitators, and inconsistent factors fell into this category. A total of thirty-eight factors from twenty-one studies were grouped into this category and could be further grouped into six subcategories (demographics, physical status, mental status, perceptions and beliefs, behavior habits, and additional factors), as detailed in Table 2.

**TABLE 2:** Five categories of factors affecting polypharmacy adherence in PLWH.

Factors	No. of studies in which a significant negative relationship was found	No. of studies in which a significant positive relationship was found	No. of studies in which no significance was found
Individual factors			
Demographics			
Older age	2	6	2
Female	3	1	4
White race		2	1
Ethnicity	2	1	1
Physical status			
BMI*			1
Poor neurocognition	1		
≥1-bed day for illness in the previous 12 months	1		
Self-reported good general health		1	
Mental status			
Depressive/anxiety symptoms	4		
Negative attitude	1		
Fear of disclosure	1		
Poor endurance	1		
Worrying about taking medicine	2		
Worrying about side effects	4		
Feeling life is disrupted	2		
Perceptions and beliefs			
Belief in the necessity of medications		2	
Belief in the effectiveness of medications		2	
Belief in the serious outcomes of non-adherence		2	
Belief in the curability of diseases		1	
Belief in the severity of diseases		1	
Belief in one’s ability to take medications consistently		1	
Behavior and experience			
Alcohol use	2		1
Illegal drug use	1		1
Smoking	1		
Cocaine use	1		
Marijuana use	1		
IVDU	2		
Restricted ADLs	1		
Medication non-compliance history	1		
Good compliance with selected medications		2	
Visiting the disease center regularly		1	
Additional factors			
Forgetfulness	1		
Too busy	1		
Previous hospitalizations	1		
Good tolerance to diseases and drugs		1	
Sexual transmission		1	
Number of stressors/stressful experiences			1
History of imprisonment			1
Condition-related factors			
Comorbidities	4		
Coinfection	1		
Detectable viral load	4		
Low CD4^+^ count	3		
AIDS diagnosis	1		
Treated with psychotropic medications	1		
ART failure	1		
Hospitalization	1		
WHO stage (early)		1	
HIV diagnosed for a long period of time		1	
Number of HIV-related diseases			1
HIV symptoms			1
Treatment-related factors			
Complex dosing regimen	5		
Pill burden	5		
Number of comedications	1		
High frequency of taking medications	1		
Stopped HIV therapy for at least 1 week	1		
Receiving a particular treatment or drug	1		
Taking NNRTI-based therapies		1	
Concomitant medication use	2	4	
Side effects of medications	5		1
Polypharmacy	3		1
Therapeutic changes	1	1	
ART treatment experience	1	1	
Healthcare provider-related factors			
Poor communication with health professionals	2		
Inadequate promotion of treatment literacy	1		
Reasonable advice from healthcare providers		1	
Supervision by healthcare providers		1	
Support from healthcare professionals		1	
Socioeconomic factors			
Poverty	5		
Taking medications at home	2		
Social isolation and stigmatization	2		
Disability grant	1		
Social support		4	
Support from family and friends		3	
Financial support		2	
Living in a high-prevalence area		1	
Education level (low)	2		3
Employment		1	2
Married			1
Informal settlement			1
Healthcare-deprived area			1

Abbreviations: ADLs, activities of daily living; AIDS, acquired immunodeficiency syndrome; ART, antiretroviral therapy; BMI, body mass index; HIV, human immunodeficiency virus; IVDU, intravenous drug use; NNRTI, non-nucleoside reverse transcriptase inhibitor; WHO, world health organization.

*Body mass index (BMI) is a person’s weight in kilograms (or pounds) divided by the square of height in meters (or feet).

Four factors, including age, sex, ethnicity, and race, fell into the demographics subcategory. Twelve studies reported inconsistent results regarding the relationships between demographics and polypharmacy adherence. The most complex relationship was between age and polypharmacy adherence. One study (O’Donnell et al., 2014) in South Africa showed that younger age was associated with good polypharmacy adherence (OR = 2.95; 95% CI = 0.65–13.42; *p* < 0.001). Another study (Gimeno-Gracia and Sanchez-Rubio-Ferrandez, 2020) in Spain reported a similar result. However, five cohort studies and one randomized controlled trial (RCT) in the United States (Cohn et al., 2002; Walkup et al., 2008; Kumar and Encinosa, 2009; Kalichman et al., 2015; Casaletto et al., 2016) and Canada (Krentz and Gill, 2016) found that older age was associated with better polypharmacy adherence (OR: 1.48; 95% CI: 1.05–2.10; *p* = 0.05). According to the study conducted by Zelnick et al.( 2021), the relationship between age and compliance was not significant (OR: 0.76; 95% CI = 0.32–1.77; *p* = 0.52). Different age groups have different barriers to and facilitators of polypharmacy adherence. Therefore, when evaluating barriers to polypharmacy adherence, healthcare providers should not use age as a predictor but consider the barriers that patients in a particular age group may face and the actual situation.

Mental status was an important barrier to polypharmacy adherence at the individual factor level because the number of barriers identified in this category was the highest. Seven barriers were identified in the mental status subcategory. These barriers were reported in eight studies from the United States (Cohn et al., 2002; Kumar and Encinosa, 2009; Yi et al., 2011; Kalichman et al., 2015; Casaletto et al., 2016), Switzerland (Kamal et al., 2018), Tanzania (Mangesho et al., 2014), and Ethiopia (Gebremariam et al., 2010).

The perceptions and beliefs of PLWH were major facilitators of polypharmacy adherence at the individual factor level, with the most facilitators identified in this subcategory. Six factors that had positive associations with polypharmacy adherence were identified in this subcategory. Seven studies in the United States (Eldred et al., 1998; Cohn et al., 2002; Kalichman et al., 2015), Switzerland (Kamal et al., 2018), Tanzania (Mangesho et al., 2014), South Africa (Daftary et al., 2014), and Ethiopia (Gebremariam et al., 2010) identified six facilitators.

#### 3.3.2 Condition-related factors

A total of thirteen factors were identified from ten studies in the condition-related factors category, including nine negative factors, two positive factors, and two irrelevant factors; these factors are presented in Table 2.

The most common factors hindering the maintenance of a high level of polypharmacy adherence were comorbidities or coinfections (*n* = 5), a detectable viral load (*n* = 4), and a lower CD4^+^ count (*n* = 3).

The influence of comorbidities on polypharmacy adherence lies mainly in the number, type, and severity of comorbidities (Krentz and Gill, 2016; Borrego et al., 2018; Manzano-García et al., 2018; Gimeno-Gracia and Sanchez-Rubio-Ferrandez, 2020). Most studies showed that comorbidities could lead to a decrease in adherence to one or more medications, especially for patients with psychiatric disorders (Walkup et al., 2008; Kumar and Encinosa, 2009). Although Encinosa and colleagues found that the number of HIV-related diseases for which medicine was taken was not associated with polypharmacy adherence, they acknowledged the impact of non-HIV comorbidities on adherence (Kumar and Encinosa, 2009). The severity of comorbidities and the perceptions of disease in PLWH will affect their judgment when receiving treatment, leading to different attitudes toward treatments for different diseases, thus causing them to give priority to some medications over others. Gebremariam et al. (2010) showed that in Ethiopia, HIV-TB coinfection caused decreased polypharmacy adherence, as some PLWH stopped TB treatment and continued ART after weighing the benefits and costs associated with the disease.

Early-stage HIV and having an HIV diagnosis for a longer period of time were facilitators of maintaining a high level of polypharmacy adherence (Kalichman et al., 2015; Abdu and Walelgn, 2021). Kalichman *et al.* showed that in Ethiopia, having been diagnosed with HIV for a longer period of time was associated with greater adherence to both ART (r: 0.20; *p* ≤ 0.01) and psychotropic therapy (r: 0.30; *p* ≤ 0.05) among PLWH (Kalichman et al., 2015).

The numbers of HIV-related diseases and HIV symptoms were not found to be significantly associated with polypharmacy adherence in two separate studies (Kumar and Encinosa, 2009; Kalichman et al., 2015). Kalichman et al. (2015) found no significant association between HIV symptoms and adherence to ART (r: 0.01; *p* > 0.06) and psychiatric medications (r: 0.10; *p* > 0.06) in PLWH in the United States.

#### 3.3.3 Treatment-related factors

A total of twelve factors in the treatment-related factors category were identified from twenty-one studies, including six negative factors, one positive factor, and five inconsistent factors, as shown in Table 2.

The most common treatment-related factors that hindered a high level of polypharmacy adherence were complex dosing regimens (Kumar and Encinosa, 2009; Daftary et al., 2014; Jimenez Galan et al., 2016; Manzano-García et al., 2018; Gimeno-Gracia and Sanchez-Rubio-Ferrandez, 2020) and pill burden (Cohn et al., 2002; Gebremariam et al., 2010; Daftary et al., 2014; Mangesho et al., 2014; Yager et al., 2017).

Only one facilitator was identified under this category. Cantudo-Cuenca and colleagues found that PLWH undergoing non-nucleoside reverse transcriptase inhibitor (NNRTI)-based therapy presented better adherence levels with the concurrent use of comedications (31.3% vs. 41.9%. *p* = 0.01) (Cantudo-Cuenca et al., 2014).

Five factors with inconsistent findings were identified under this category. Of these factors, the most controversial factor was the concomitant use of medications (Walkup et al., 2008; Kumar and Encinosa, 2009; Calvo-Cidoncha et al., 2015; Ayele et al., 2016; Saravolatz et al., 2019; Abdu and Walelgn, 2021). Calvo-Cidoncha et al. (2015) and Abdu and Walelgn (2021) found that the addition of anti-HCV and anti-tuberculosis therapies to ART reduced the compliance of PLWH undergoing ART. However, three studies showed that the use of antidepressants, anxiolytics, and antipsychotics could improve polypharmacy compliance in PLWH with spiritual diseases (Walkup et al., 2008; Kumar and Encinosa, 2009; Saravolatz et al., 2019). Ayele et al. (2016) also found a similar result.

The second most common factor with inconsistent findings in this category was the side effects of medications. The side effects of medications, including drug–drug interactions and adverse drug reactions, were found to be barriers to maintaining high levels of polypharmacy adherence in five studies in Spain (Cantudo-Cuenca et al., 2014; Gimeno-Gracia and Sanchez-Rubio-Ferrandez, 2020), Canada (Krentz and Gill, 2016), South Africa (Daftary et al., 2014), and Ethiopia (Gebremariam et al., 2010). Kalichman et al. (2015) showed that medication side effects had no statistically significant correlation with adherence to ART (r: 0.01; *p* > 0.06) or psychiatric disorders (r: 0.12; *p* > 0.06) in PLWH. However, we feel this may be the exception rather than the rule, as most other studies have found that medication side effects were associated with non-compliance.

The third most common factor with inconsistent findings was polypharmacy. A study conducted in Spain by Gimeno-Gracia and Sanchez-Rubio-Ferrandez (2020) found that polypharmacy (defined as the simultaneous use of six or more medications) was a barrier to compliance. Studies conducted by Krentz and Gill (2016) in Canada and by Cantudo-Cuenca et al. (2014) in Spain showed consistent results, although they defined polypharmacy as the simultaneous use of five or more different types of medications daily. Another study conducted in Thailand, which defined polypharmacy as the simultaneous use of five or more non-ART drugs, showed no significant association between polypharmacy and poor adherence (Khawcharoenporn and Tanslaruk, 2020).

#### 3.3.4 Healthcare provider-related factors

Five factors in this category were identified from three qualitative studies and are shown in Table 2. Poor communication with healthcare providers and inadequate promotion of treatment literacy were barriers to maintaining a high level of polypharmacy adherence in PLWH (Gebremariam et al., 2010; Daftary et al., 2014). Reasonable advice, supervision, and support from healthcare providers were facilitators of maintaining a high level of polypharmacy adherence in PLWH (Gebremariam et al., 2010; Daftary et al., 2014; Mangesho et al., 2014).

#### 3.3.5 Socioeconomic factors

As shown in Table 2, thirteen factors from eighteen studies were identified in this category, including four barriers, four facilitators, two inconsistent factors, and three factors that were not significantly related.

Zelnick and colleagues found that PLWH who received a disability grant at baseline were less likely to maintain a high level of polypharmacy adherence than those who did not receive a disability grant (OR: 3.76; 95% CI = 1.20–11.86; *p* = 0.02) (Zelnick et al., 2021). However, we believe that the PLWH who received disability grants were disabled and thus had lower polypharmacy adherence rather than poor adherence due to receiving the grant. This is because one study showed that financial constraints reduce adherence to polypharmacy (Siefried et al., 2018).

Yi et al. (2011) found that some government-funded programs and agencies, such as the AIDS Drug Assistance Program, Medicare, and Medicaid, could promote polypharmacy adherence in the United States.

Education level and employment were two factors in this category with inconsistent results. Two studies found that a high level of education was associated with better polypharmacy adherence (Daftary et al., 2014; O’Donnell et al., 2014). However, three studies in the United States showed no significant association between education level and polypharmacy adherence (Yi et al., 2011; Kalichman et al., 2015; Zelnick et al., 2021).

#### 3.3.6 Effect of interventions on polypharmacy adherence

In addition to the five categories mentioned above, two other interventions were effective in promoting polypharmacy adherence in PLWH. Monroe et al. (2018) found that a pictorial aid intervention (a photographic representation of the medications, indications, and dosing schedule) could improve polypharmacy adherence in PLWH compared with a standard clinic visit discharge medication list. Morillo-Verdugo and colleagues (Morillo-Verdugo et al., 2021) found that a pharmaceutical care intervention based on the capacity, motivation, and opportunity (CMO) methodology could improve primary and secondary adherence to concomitant medications and ART in PLWH.

## 4 Discussion

Similar to previous studies, personal factors play an important role in medication adherence in PLWH (Vervoort et al., 2007). Understanding past and current compliance of PLWH with other treatments can help predict their medication adherence in the case of polypharmacy. Additionally, interventions to improve the knowledge and motivation of PLWH to take their medications will promote compliance with multiple medications (Monroe et al., 2018; Morillo-Verdugo et al., 2021). The difference is that adherence to different medications may be different even among the same PLWH. Compliance with ART tends to be higher than that with concomitant medications in PLWH (Daftary et al., 2014; Kamal et al., 2018; O'Donnell et al., 2014; Kalichman et al., 2015). Therefore, when assessing the adherence of PLWH, healthcare professionals need to focus on individual patient factors and assess their adherence to different medications.

However, even within the same PLWH, the barriers and facilitators that affect adherence to different treatment medications are not identical. Yager et al. (2017) showed that the use of single-tablet regimens (STRs) was associated with optimal adherence to ART medications but was not directly associated with adherence to non-ART medications. Yi et al. (2011) showed that AIDS Drug Assistant Program (ADAP) enrollment had a better effect on ART medication adherence than on hypertensive medication adherence. Therefore, when assessing barriers to polypharmacy adherence in PLWH, it is necessary to evaluate the adherence to each prescribed drug individually as well as to all drugs collectively.

We found that unlike in PLWH undergoing only ART or the general population, in patients with polypharmacy, non-ART medications affect adherence to ART drug therapy and *vice versa* (Monroe et al., 2018; Siefried et al., 2018). The factors influencing adherence to polypharmacy in HIV-infected patients are more complex than those in the general population. Previous studies have found that concomitant medication use decreases patient medication adherence, but this study reported inconsistent factors (Mills et al., 2006; Vervoort et al., 2007). The effect of polypharmacy and concomitant medication use on medication compliance depends on the type, quantity, and regimen of the medications (Kumar and Encinosa, 2009; Manzano-García et al., 2018). On the one hand, several studies have shown that the use of antidepressant and anti-anxiety drugs significantly increased the compliance of PLWH taking ART and psychotropic medications (Walkup et al., 2008; Kumar and Encinosa, 2009; Kalichman et al., 2015; Saravolatz et al., 2019). On the other hand, the use of multidrug therapy or concomitant drugs can hinder compliance by increasing the complexity of the medication regimen, pill burden, and risk of drug–drug interactions in PLWH (Calvo-Cidoncha et al., 2015; Abdu and Walelgn, 2021). For example, a study conducted in the United States by Calvo-Calvo-Cidoncha et al. (2015) showed that the addition of anti-hepatitis C (HCV) virus therapy to ART reduced compliance with ART drugs because the addition of anti-HCV therapy greatly increased the complexity of the overall medication regimen. Medications should be evaluated separately, and an overall medication evaluation should be conducted simultaneously to prevent inappropriate polypharmacy use from reducing polypharmacy adherence.

To our knowledge, this review is the first comprehensive synthesis of studies exploring factors associated with polypharmacy adherence in PLWH. This model can help healthcare providers and caregivers of PLWH predict polypharmacy adherence from many aspects, thus helping them provide appropriate interventions at reasonable times. Therapeutic decisions at the hospital, community and administrative levels regarding the medication burden of PLWH should be more nuanced, considering both treatment factors and individual affordability and compliance with the medication burden. However, the conclusions and significance of different studies vary greatly from region to region, so medical workers in different regions should consider the application of various interventions based on the actual local situation.

Although this is the first review of all factors affecting polypharmacy adherence in PLWH, there are still several limitations. First, a weakness of this study is that it was a scoping review and not a meta-analysis of the effects of various barriers and facilitators. It is difficult to determine the degree of influence of different factors due to the small number of studies and considerable heterogeneity among studies. Second, it is difficult to set a prescribed compliance threshold for the integration of all included studies, and instead, we used the optimal compliance thresholds of the individual studies. Third, we excluded studies of patients under the age of 18 years when searching the literature, which may have resulted in missing data from some of the studies that included adults.

Several points should be noted when interpreting our results. Most studies only focus on specific comorbidities or concomitant medication use in PLWH, so there is no overall situation of polypharmacy in PLWH. The included studies only focused on certain chronic complications or coinfections and lacked research on the overall combined treatment of PLWH. This gives researchers and health providers a hint that the creation of a comorbidity network to better understand comorbidities and polypharmacy in PLWH may be possible (Zhu et al., 2021). The lack of a consistent definition of polypharmacy introduces difficulties in identifying the relevant factors not only to research but also to users. Future research needs to focus on developing a unified, relevant definition of polypharmacy. Most of the studies included in this review were observational studies, and there is a lack of research on interventions in PLWH.

## 5 Conclusion

Eighty factors associated with polypharmacy adherence among PLWH were identified and grouped into five major categories, including individual factors, treatment-related factors, condition-related factors, healthcare provider-related factors, and socioeconomic factors. Healthcare providers can make decisions based on the five categories of relevant factors described in this paper when developing interventions to enhance polypharmacy adherence in PLWH. We recommend evaluating medications separately and conducting an overall evaluation of medications at the same time to prevent inappropriate polypharmacy use that could decrease polypharmacy adherence. Hospital-, community- and administrative-level therapeutic decision-making regarding the medication burden of PLWH should be more nuanced, taking treatment factors and individual affordability and polypharmacy adherence into consideration.
