# Supplementary material for: Barriers and facilitators to maintaining a high level of polypharmacy adherence in people living with HIV: A scoping review
Source: Front Pharmacol. 2023 Mar 2;14:1013688. doi: 10.3389/fphar.2023.1013688 (PMC10017548; doi:10.3389/fphar.2023.1013688)
Supplement: Supplementary file 1 [file DataSheet1.PDF]

Supplementary Material

1 Supplementary file

PubMed

Search time: 2021-10-25 16:10

Search	Query	Items found
#1	"HIV"[MeSH Terms] OR "HIV Infections"[MeSH Terms] OR "Acquired Immunodeficiency Syndrome"[MeSH Terms]	331556
#2	"hiv"[Title/Abstract] OR "hiv infect*"[Title/Abstract] OR "AIDS"[Title/Abstract] OR "PLWH"[Title/Abstract] OR "PLWHA"[Title/Abstract] OR "human immunodeficiency virus"[Title/Abstract] OR "human immunedeficiency virus"[Title/Abstract] OR "human immune deficiency virus"[Title/Abstract] OR ("human immun*"[Title/Abstract] AND "deficiency virus"[Title/Abstract]) OR "acquired immune deficiency syndrome"[Title/Abstract] OR ("acquired immun*"[Title/Abstract] AND "deficiency syndrome"[Title/Abstract])	428930
#3	#1 OR #2	468199
#4	"Medication Adherence"[MeSH Terms] OR "Patient Compliance"[MeSH Terms] OR "Treatment Adherence and Compliance"[MeSH Terms]	262406
#5	(medication[Title/Abstract] OR drug therapy[Title/Abstract] OR pharmacotherapy[Title/Abstract] OR pharmacon[Title/Abstract] OR treatment[Title/Abstract] OR therapy[Title/Abstract] OR drug[Title/Abstract] OR medicine[Title/Abstract] OR prescription[Title/Abstract] OR pill[Title/Abstract] OR tablet[Title/Abstract] OR remedy[Title/Abstract]) AND (adhere*[Title/Abstract] OR complian*[Title/Abstract] OR non adheren*[Title/Abstract] OR nonadheren*[Title/Abstract] OR non complian*[Title/Abstract] OR noncomplian*[Title/Abstract] OR persistence[Title/Abstract] OR concordan*[Title/Abstract] OR insist*[Title/Abstract] OR	212278

	obedience[Title/Abstract] OR refusal[Title/Abstract])	
#6	#4 OR #5	430710
#7	"Polypharmacy"[MeSH Terms] OR "drug therapy, combination"[MeSH Terms] OR "Drug Combinations"[MeSH Terms]	433827
#8	"multiple medicat*"[Title/Abstract] OR "multidrug"[Title/Abstract] OR "drug polytherapy"[Title/Abstract] OR "polymedicine"[Title/Abstract] OR "multiple drug regimen"[Title/Abstract] OR "combination therapy"[Title/Abstract]	126127
#9	#7 OR #8	532705
#23	#3 AND #6 AND #9 Filters: Chinese, English, from 1996/1/1–2021, Adults:19+years	3150
#24	"comedications"[Title/Abstract] OR "co-medications"[Title/Abstract] OR "concomitant medications"[Title/Abstract] OR "polymedication"[Title/Abstract] OR "coprescriptions"[Title/Abstract]	3078
#25	#3 AND #6 AND #24 Filters: Chinese, English, from 1996/1/1–2021, Adults:19+years	21
#26	#23 OR #25	3171

EMBASE (ovid)

Search time: 2021-10-25 17:20

**Search**	**Query**	**Items found**
1	(HIV or Acquired Immunodeficiency Syndrome).hw.	31243

2	(hiv or AIDS or PLWH or PLWHA or acquired immunodeficiency syndrome).ti,ab.	521151
3	1 or 2	524768
4	exp medication compliance/	37691
5	exp patient compliance/	170616
6	4 or 5	170616
7	(medication or 'drug therapy' or pharmacotherapy or pharmacon or treatment or therapy or drug or medicine or prescription or pill or tablet or remedy).ti,ab.	9397758
8	('adhere$' or 'complian$' or 'non adheren$' or 'nonadheren$' or 'non complian$' or 'noncomplian$' or persistence or 'concordan$' or 'insist$' or obedience or refusal).ti,ab.	763094
9	7 and 8	353080
10	6 or 9	444460
11	exp polypharmacy/	19283
12	combination drug therapy/	16886
13	11 or 12	35991
14	(('multiple adj3 medicat$') or 'multidrug$' or 'drug polytherapy' or polymedicine or 'multiple drug regimen' or 'combination therapy').ti,ab.	158755
15	13 or 14	190618
16	3 and 10 and 15	1024
17	Limit 16 to (Chinese or English, and yr="1996 -Current")	944
18	(comedications or co-medications or concomitant medications or polymedication or coprescriptions).ti,ab.	6338

19	3 and 10 and 18	63
20	Limit 19 to (Chinese or English, and yr="1996 -Current")	63
21	17 or 20	1007

CINAHL (EBSCO)

**Search**	**Query**	**Items found**
S1	(MH "Human Immunodeficiency Virus") OR (MH "Acquired Immunodeficiency Syndrome") OR (MH "HIV Infections")	87031
S2	TI (hiv or aids or PLWH or PLWHA or 'human immunodeficiency virus' or 'human immune-deficiency virus' or 'human immunedeficiency virus' or 'acquired immune-deficiency syndrome' or 'acquired immunedeficiency syndrome' or 'acquired human immunodeficiency syndrome') OR AB (hiv or aids or PLWH or PLWHA or 'human immunodeficiency virus' or 'human immune-deficiency virus' or 'human immunedeficiency virus' or 'acquired immune-deficiency syndrome' or 'acquired immunedeficiency syndrome' or 'acquired human immunodeficiency syndrome')	123597
S3	S1 OR S2	123597
S4	(MM "Medication Compliance") OR (MH "Patient Compliance")	44441
S5	TI (medication or 'drug therapy' or pharmacotherapy or pharmacon or treatment or therapy or drug or medicine or prescription or pill or tablet or remedy) OR AB (medication or 'drug therapy' or pharmacotherapy or pharmacon or treatment or therapy or drug or medicine or prescription or pill or tablet or remedy)	1608088
S6	TI ('adhere*' or 'complian*' or 'non adheren*' or 'nonadheren*' or 'non complian*' or 'noncomplian*' or persistence or 'concordan*' or 'insist*' or obedience or refusal) OR AB ('adhere*' or 'complian*' or 'non adheren*'	140822

	or 'nonadheren*' or 'non complian*' or 'noncomplian*' or persistence or 'concordan*' or 'insist*' or obedience or refusal)	
S7	S5 AND S6	67188
S8	S4 OR S7	67188
S9	(MM "Polypharmacy")	2168
S10	TI ('multiple medicat*' or 'multidrug*' or 'drug polytherapy' or polymedicine or 'multiple drug regimen' or 'combination * therapy') OR AB ('multiple medicat*' or 'multidrug*' or 'drug polytherapy' or polymedicine or 'multiple drug regimen' or 'combination * therapy')	21374
S11	S9 OR S10	21374
S12	S3 AND S8 AND S11	286
S13	Limit 13 to (English or Chinese, and publish date:1996-2021)	281
S14	TI (comedications or co-medications or concomitant medications or polymedication or coprescriptions) OR AB (comedications or co-medications or concomitant medications or polymedication or coprescriptions)	2006
S15	S3 AND S8 AND S14	16
S16	Limit 13 to (English or Chinese, and publish date:1996-2021)	16
S17	S13 OR S16	297

Search time: 2021-10-22 18:00

Web of Science

Search time: 2021-10-23 08:00

**Search**	**Query**	**Items found**
#1	TS=(HIV OR Acquired Immunodeficiency Syndrome OR AIDS OR PLWH OR PLWHA OR human immunodeficiency virus OR human immuno-deficiency virus OR human immunodeficiency virus OR human immune-deficiency virus OR acquired immune-deficiency syndrome OR acquired immunodeficiency syndrome OR acquired immunodeficiency syndrome OR acquired immuno-deficiency syndrome)	2019142

#2	TS=(medication or 'drug therapy' or pharmacotherapy or pharmacon or treatment or therapy or drug or medicine or prescription or pill or tablet or remedy or patient)	31872408
#3	TS=('adhere*' or 'complian*' or 'non adheren*' or 'nonadheren*' or 'non complian*' or 'noncomplian*' or persistence or 'concordan*' or 'insist*' or obedience or refusal)	1856891
#4	(#3) AND #2	709628
#5	TS=(polypharmacy or 'multiple medicat*' or 'multidrug*' or 'drug polytherapy' or polymedicine or 'multiple drug regimen' or 'combination * therapy')	975978
#6	((#1) AND #4) AND #5	7802
#7	((#1) AND #4) AND #5<br><br>and 2021 or 2020 or 2019 or 2018 or 2017 or 2016 or 2015 or 2 014 or 2013 or 2012 or 2010 or 2011 or 2009 or 2008 or 2007 o r 2006 or 2005 or 2004 or 2003 or 2002 or 2001 or 2000 or 199 9 or 1997 or 1998 or 1996 (Publication Years)	7613
#8	TS=(comedications or co-medications or concomitant medications or polymedication or coprescriptions)	14289
#9	((#1) AND #4) AND #8	165
#10	((#1) AND #4) AND #8<br><br>and 2021 or 2020 or 2019 or 2018 or 2017 or 2016 or 2015 or 2 014 or 2013 or 2012 or 2010 or 2011 or 2009 or 2008 or 2007 o r 2006 or 2005 or 2004 or 2003 or 2002 or 2001 or 2000 or 199 9 or 1997 or 1998 or 1996 (Publication Years)	162
#11	#7 OR #10	7775

ProQuest Dissertations and Theses Global

Search time: 2021-10-23 14:00

Search	Query	Items found
S1	ti(HIV OR Acquired Immunodeficiency Syndrome OR AIDS OR PLWH OR PLWHA OR human immunodeficiency virus OR human immuno-deficiency virus OR human immunodeficiency virus OR human immune-deficiency virus OR acquired immune-deficiency syndrome OR acquired immunodeficiency syndrome OR acquired immunodeficiency syndrome OR acquired immuno-deficiency syndrome) OR ab(HIV OR Acquired Immunodeficiency Syndrome OR AIDS OR PLWH OR PLWHA OR human immunodeficiency virus OR human immuno-deficiency virus OR human immunodeficiency virus OR human immune-deficiency virus OR acquired immune-deficiency syndrome OR acquired immunodeficiency syndrome OR acquired immunodeficiency syndrome OR acquired immuno-deficiency syndrome)	96373
S2	ti(medication or 'drug therapy' or pharmacotherapy or pharmacon or treatment or therapy or drug or medicine or prescription or pill or tablet or remedy or patient) OR ab(medication or 'drug therapy' or pharmacotherapy or pharmacon or treatment or therapy or drug or medicine or prescription or pill or tablet or remedy or patient)	588596
S3	ti('adhere*' or 'complian*' or 'non adheren*' or 'nonadheren*' or 'non complian*' or 'noncomplian*' or persistence or 'concordan*' or 'insist*' or obedience or refusal) OR ab('adhere*' or 'complian*' or 'non adheren*' or 'nonadheren*' or 'non complian*' or 'noncomplian*' or persistence or 'concordan*' or 'insist*' or obedience or refusal)	103876
S4	S2 AND S3	24560
S5	ti(polypharmacy or 'multiple medicat*' or 'multidrug*' or 'drug polytherapy' or polymedicine or 'multiple drug regimen' or 'combination therapy') OR ab(polypharmacy or 'multiple medicat*' or 'multidrug*' or 'drug polytherapy' or polymedicine or 'multiple drug regimen' or 'combination therapy')	12956
S6	S1 AND S4 AND S5 (1996-01-01 to 2021-10-23)	217
S7	ti(comedications or co-medications or concomitant medications or polymedication or coprescriptions) OR	212

	ab(comedications or co-medications or concomitant medications or polymedication or coprescriptions)	
S8	S1 AND S4 AND S5 (1996-01-01 to 2021-10-23)	1
S9	S6 OR S8	218

Cochrane Library

Search time: 2021-10-23 22:36

**Search**	**Query**	**Items found**
#1	MeSH descriptor: [HIV] explode all trees	3174
#2	(HIV OR Acquired Immunodeficiency Syndrome OR AIDS OR PLWH OR PLWHA OR human immunodeficiency virus OR human immuno-deficiency virus OR human immunodeficiency virus OR human immune-deficiency virus OR acquired immune-deficiency syndrome OR acquired immunodeficiency syndrome OR acquired immunodeficiency syndrome OR acquired immuno-deficiency syndrome):ti,ab,kw	46356
#3	#1 OR #2	46356
#4	MeSH descriptor: [Medication Adherence] explode all trees	2580
#5	(medication or 'drug therapy' or pharmacotherapy or pharmacon or treatment or therapy or drug or medicine or prescription or pill or tablet or remedy or patient):ti,ab,kw	1453358
#6	('adhere*' or 'complian*' or 'non adheren*' or 'nonadheren*' or 'non complian*' or 'noncomplian*' or persistence or 'concordan*' or 'insist*' or obedience or refusal):ti,ab,kw	127456
#7	#5 AND #6	117316
#8	#4 OR #7	117316

#9	MeSH descriptor: [Polypharmacy] explode all trees	227
#10	('multiple medicat*' or 'multidrug*' or 'drug polytherapy' or polymedicine or 'multiple drug regimen' or 'combination therapy'):ti,ab,kw	188509
#11	#9 OR #10	188662
#12	#3 AND #8 AND #11	1561
#13	(comedications or co-medications or concomitant medications or polymedication or coprescriptions):ti,ab,kw	6589
#14	#3 AND #8 AND #13	70
#15	#12 OR #14	1631

CNKI*

Search time: 2021-10-23 15:40

**Search**	**Query**	**Items found**
#1	(title, abstract, and keywords)% Human Immunodeficiency Virus + Acquired Immunodeficiency Syndrome + HIV + AIDS	811504
#2	(title, abstract, and keywords)% drug therapy compliance + medication compliance + medicine compliance + treatment compliance	89801
#3	(title, abstract, and keywords)% polypharmacy + multi-drug treatment + multi-drug combinations	372
#4	#1 AND #2 AND #3	0

*The search strategy was translated into English.

Wanfang*

Search time: 2021-10-23 16:05

**Search**	**Query**	**Items found**
#1	Topic: (Human Immunodeficiency Virus or Acquired Immunodeficiency Syndrome or HIV or AIDS)	672319
#2	Topic: (drug therapy compliance or medication compliance or medicine compliance or treatment compliance)	101573
#3	Topic: (polypharmacy or multi-drug treatment or multi-drug combinations)	571713
#4	(#1 AND #2 AND #3) and Date: 1996-2021	520

*The search strategy was translated into English.

SinoMed

Search time: 2021-10-23 16:30

**Search**	**Query**	**Items found**
#1	Title: ("Human Immunodeficiency Virus" OR "Acquired Immunodeficiency Syndrome" OR HIV OR AIDS) OR Abstract: ("Human Immunodeficiency Virus" OR "Acquired Immunodeficiency Syndrome" OR HIV OR AIDS)	52820
#2	Title: (drug therapy compliance OR medication compliance OR medicine compliance OR treatment compliance) OR Abstract: (drug therapy compliance OR medication compliance OR medicine compliance OR treatment compliance)	36581
#3	Title: (polypharmacy OR multi-drug treatment OR multi-drug combinations) OR Abstract: (polypharmacy OR multi-drug treatment OR multi-drug combinations)	216
#4	#1 AND #2 AND #3	0

*The search strategy was translated into English.

2 Supplementary Table

Barriers and facilitators to polypharmacy adherence

Study, year	Barriers found to significantly affect adherence	Facilitators found to significantly affect adherence	Determinants found to not significantly affect adherence (other key findings)
Zelnick, 2021		Social grant (*OR: 3.80; 95% CI: 1.21-11.94; P=0.02*)<br><br>Baseline viral load (*OR: 2.21; 95% CI: 1.03-4.74; P=0.04*)	Age (>36) (*OR: 0.76; 95% CI: 0.32-1.77; P=0.52*)<br><br>Gender (female) (*OR: 2.07; 95% CI: 0.72-5.95; P=0.18*)<br><br>Education level (*OR: 0.92; 95% CI: 0.35-2.42; P=0.86*)<br><br>Body mass index (BMI) (*OR: 0.95; 95% CI: 0.41-2.21; P=0.90*)<br><br>History of alcohol use (*OR: 1.76; 95% CI: 0.65-4.79; P=0.27*)<br><br>History of imprisonment (*OR: 1.79; 95% CI: 0.53-6.05; P=0.35*)<br><br>Informal settlement (*OR: 0.36; 95% CI: 0.08-1.66; P=0.19*)
Abdu, 2021		Not take anti-tuberculosis medication collaterally (A*OR=2.27; 95% CI=1.26-4.10; P=0.007)*<br><br>ART treatment more than 24 months (A*OR=3.67; 95% CI=1.32-10.17; P=0.013)*<br><br>Baseline WHO stage (stage I)<br><br>(A*OR=2.19; 95% CI=1.12-4.31; P=0.023)*	Initiation of antiretroviral therapy at the later stage of the disease and taking anti-tuberculosis medication concomitantly were negatively associated with adherence.
Morillo-Verdugo, 2021		CMO methodology (capacity, motivation, and opportunity)	
Gimeno-Gracia, 2020	Age (older)<br><br>Polypharmacy (≥6 AIs)<br><br>Medication regimen complexity<br><br>Drug–drug interactions<br><br>Comorbidities		

Khawcharoen porn, 2020			Polypharmacy (≥5 non-ART drugs)
Saravolatz, 2019	Stopped HIV therapy (*OR=6.33; 95% CI=1.8-22.0; P=0.004)*	Psychiatric medications use (anti-depressant, anxiolytic, or anti-psychotic) (*OR=0.28; 95% CI=0.08-0.99; P=0.05)*	Age (P=0.66)<br><br>Gender (P=0.52)<br><br>Race (P=0.29)<br><br>Healthcare-deprived area (P=0.31)
Manzano-García, 2018	Comorbidities (*OR: 1.04-1.57; P<0.021*)<br><br>Detectable viral load (≥20 copies/ml) (*OR: 1.11-4.51; P=0.023*)<br><br>High MRCI (*OR: 1.14-1.26; P<0.0001*)		Gender (*OR: 0.88-2.25; P=0.154*)
Siefried, 2018	Require financial support (*AOR=27.8; 95% CI=1.8-440; P=0.018*)<br><br>Financial constraints (*AOR=11.1; 95% CI=1.9-114; P=0.042*)<br><br>Day in bed for illness (AOR=14.0; *95% CI=1.2-163; P=0.035*)	Good self-reported general health (*AOR=14.1; 95% CI=1.4-141; P=0.025*)	
Monroe, 2018		A pictorial aid intervention (a photographic representation of the medications, the indications, and the dosing schedule)	
Borrego, 2018	**Concomitant medications:**<br><br>Comedications numbers (*OR=1.18; 95% CI=1.08-1.28; P<0.001)*<br><br>Number of comorbidities (OR=1.34; 95% CI=1.12-1.61; P=0.001)<br><br>Patients treated with psychotropic drugs (OR=1.67; 95% CI=1.05-2.66; P=0.001)		
Kamal, 2017		Higher necessity belief<br><br>Lower concern belief	A higher percentage of patients reported adherent to ART compared with co-treatments.
Yager, 2017		STR (*36.4% vs. MTR 11.3%; P<0.001*)†<br><br>Adherence to ART medications (AOR*: 2.30, 95% CI: 1.57-3.38; P<0.001*)	Optimal adherence to ART medications was independently associated with optimal adherence to non-ART medications.
Krentz, 2016	Polypharmacy (≥5 daily medications)<br><br>(*36.8% vs. 30.0%; P<0.05*)†		

	Gender (female) (*37.5% vs. 30.5%; P<0.05*)† <br>IVDU (50.7*% vs. 29.6%; P<0.05*)† <br>CD4 count (≤200/mm^3) (*52.2% vs. 29.1%; P<0.05*)† <br>Age (≤50) (*55.3% vs. 41.7%; P<0.05*)† <br>ADE (46.0*% vs. 29.5%; P<0.05*)† <br>Therapeutic changes (24.3*% vs. 16%; P<0.05*)† <br>Drug–drug interactions <br>ART failure		
Jiménez Galán, 2016	Pharmacotherapeutic complexity index		
Casaletto, 2016	Age (younger) <br>Depressive symptoms <br>Negative attitudes <br>Poor neurocognition <br>Previous hospitalizations		Both psychiatric and neurocognitive factors contribute to poorer psychotropic therapy adherence among PLWH with serious mental illness.
Ayele, 2016		Concomitant ART or CPT (*OR=2.66; 95% CI=1.15-6.17; P=0.041*)	PLWH receiving ART were more likely to adherent IPT
Kalichman, 2015	**ART** <br>High viral load (*r: -0.40*; *P≤0.01*) <br>**Psychiatric** <br>High viral load (*r:* -0.45; *P≤0.01*) <br>Depression (CESD) (r: -0.21; *P≤0.01*) <br>Higher medication concerns beliefs (*r:* -0.27; *P≤0.01*)	**ART** <br>Age (older) (*r: 0.35*; *P≤0.01*) <br>High income (*r: 0.24*; *P≤0.01*) <br>HIV diagnosed for more years (*r: 0.20*; *P≤0.01*) <br>Higher CD4 count (*r: 0.22*; *P≤0.01*) <br>Social support (*r: 0.29*; *P≤0.01*) <br>Higher medication necessity beliefs (*r: 0.32*; *P≤0.01*) <br>**Psychiatric** <br>Age (older) (*r:* 0.32; *P≤0.01*) <br>High income (*r:* 0.19; *P≤0.05*) <br>HIV diagnosed for more years	Gender (r=0.17/0.13; P>0.06) <br>Ethnicity (r=-0.09/-0.08; P>0.06) <br>Education (r=0.03/0.14; P>0.06) <br>Employment (r=0.05/0.8; P>0.06) <br>Drug use (r=-.002/-.02; P>0.06) <br>HIV symptoms (r=-0.01/-0.10; P>0.06) <br>Medication side effects (r=-0.01/-0.12; P>0.06) <br>Number of stressors (r: 0.02/-0.05; P>0.06) <br>Stress experience (r: -0.02/-0.09; P>0.06) <br>Audit score (r: 0.08/0.03; P>0.06)

		(*r:* 0.30; *P≤0.05*)<br><br>Higher CD4 count (*r:* 0.29; *P≤0.01*)<br><br>Social support (*r:* 0.30; *P≤0.01*)<br><br>Higher medication necessity beliefs (*r:* 0.27; *P≤0.01*)<br><br>ART adherence (r: 0.76; P≤0.01)	
Calvo-Cidoncha, 2015	Addition of anti-HCV therapy to ART<br><br>(before 79.2% *vs.* after 69.8%; P<0.001)		
Cantudo-Cuenca, 2014	IVDU (*OR=0.56; 95% CI=0.35-0.90; P≤0.05)*<br><br>Previous treatment with ART (*OR=0.09; 95% CI=0.04-0.24; P≤0.05)*<br><br>No treatment changes (*OR=0.12; 95% CI=0.05-0.31; P<0.001)*<br><br>High risk of drug-related problems (*OR=0.38; 95% CI=0.23-0.63; P<0.001)*<br><br>Polypathology (*OR=0.35; 95% CI=0.21-0.57; P<0.001)*<br><br>Polypharmacy (≥5 AIs) (*OR=0.36; 95% CI=0.21-0.61; P<0.001)*<br><br>AIDS diagnosis (*OR=0.50; 95% CI=0.34-0.76; P=0.001)*<br><br>Hospital admission (*OR=0.40; 95% CI=0.22-0.73; P=0.003)*	Sexual transmission (*OR=0.40; 95% CI=0.26-0.60; P<0.001)*<br><br>Undetectable viral load (*OR=0.47; 95% CI=0.32-0.70; P<0.001)*<br><br>High $CD4^{+}$ counts (*OR=1.00; 95% CI=1.00-1.00; P=0.031)*<br><br>Taking NNRTI-based therapies (*OR=0.56; 95% CI=0.36-0.87; P=0.01)*	
O'Donnell, 2014		**Dual adherence**<br><br>High education attainment (*OR=5.39; 95% CI=1.03-28.25; P<0.001)*<br><br>Gender (female) (*OR=4.68; 95% CI=1.11-19.68; P<0.001)*<br><br>Age (younger) (*OR=2.95; 95% CI=0.65-13.42; P<0.001)*<br><br>**ART**<br><br>Adherence to anti-TB medications (*OR=21.0; 95% CI=2.38-184.89; P=0.006)*	Medication adherence was significant higher for ART than for TB medications; Short course treatment regimens for drug-resistant TB with lower pill burden may increase adherence.

Mangesho, 2014	Incorrect perceptions of drug strength<br><br>Worry about side effects<br><br>Pill burden<br><br>Taking medications at home	Attending the HIV center regularly<br><br>Beliefs in medication effectiveness<br><br>Beliefs in the serious outcomes of nonadherence<br><br>Health workers' advice	Perceptions of drug strength appeared to compel some people not enrolled in the clinical study to take the drugs at separate times to prevent harm to the body.
Daftary, 2014	Regimen complexity<br><br>Pill burden<br><br>Medication side effects<br><br>Poor perceived treatment outcomes<br><br>Inadequate communication<br><br>Inadequate treatment literacy promotion<br><br>Social isolation<br><br>Stigma	Greater tolerability<br><br>Commitment to ART<br><br>Provider supervision<br><br>Social support	Participants expressed a preference for ART over M/XDR ‑ TB treatment.
Kebede, 2012	**Anti-TB:**<br><br>Education status (lower)<br><br>**Global:**<br><br>Lack of money for transport<br><br>Forgetfulness		
Yi, 2011	**ART:**<br><br>Not employed (*OR=1.38, 95% CI=1.03-1.84; P=0.03*)<br><br>Depression (*OR=1.40; 95% CI=1.06-1.84; P=0.02*)<br><br>Illegal drug use (*OR=1.91, 95% CI=1.23-2.95; P=0.003*)<br><br>Alcohol use (*OR=3.75, 95% CI=1.59-8.84; P≤0.05*)<br><br>Smoking (*OR=1.59; 95% CI=1.00-2.14; P<0.001*)<br><br>Marijuana use (*OR=1.46; 95% CI=1.21-2.09; P≤0.05*)<br><br>Without ADAP enrollment (*OR=2.35, 95%*	**ART:**<br><br>Age (older) (*OR=0.74, 95% CI=0.61-0.89; P≤0.05*)<br><br>Ethnicity (Hispanic white) (*OR=0.51, 95% CI=0.29-0.92; P≤0.05*)<br><br>Ethnicity (non-Hispanic white) (*OR=0.21, 95% CI=0.10-0.44; P≤0.05*)<br><br>High income (*OR=0.70 95% CI=0.50-0.99; P≤0.05*)<br><br>**Antihypertensive:**<br><br>High income (*OR=0.20 95% CI=0.06-0.71; P≤0.05*)<br><br>Medicare *vs.* Private or other health insurance (*OR=0.22 95%*	**ART**<br><br>High level of education (*OR=0.83, 95% CI=0.60-1.14; P=0.25*)<br><br>Married, living with partner (*OR=0.81, 95% CI=0.60-1.10; P=0.17*)<br><br>**Antihypertensive:**<br><br>ADAP enrollment (*OR=2.37, 95% CI=0.93-6.03; P=0.07*)<br><br>High level of education (*OR=0.79, 95% CI=0.45-1.39; P=0.41*)<br><br>Married, living with partner (*OR=0.75, 95% CI=0.0.43-1.32; P=0.32*)

	CI=1.49-3.71; P≤0.05)<br><br>Medicaid *vs.* Private or other health insurance (*OR=1.87; 95% CI=1.25-2.78; P=0.002*)<br><br>**Antihypertensive:**<br><br>Ethnicity (non-Hispanic white) (*OR=2.36, 95% CI=0.99-5.63; P≤0.05*)<br><br>Ethnicity (not African American) (*OR=4.76, 95% CI=2.48-9.14; P<0.001*)<br><br>Smoking (*OR=1.80 95% CI=1.03-3.16; P≤0.05*)	*CI=0.08-0.62; P=0.004*)<br><br>Medicaid *vs.* Private or other health insurance (*OR=0.23 95% CI=0.09-0.60; P=0.003*)	
Gebremariam, 2010	Experiencing side effects<br><br>Pill burden<br><br>Poor communication with health professionals<br><br>Economic constraints<br><br>Lack of food<br><br>Stigma<br><br>Fear of disclosure<br><br>HIV TB coinfection	Beliefs in the curability of TB<br><br>Beliefs in the severity of TB<br><br>Family support<br><br>Health professionals support	Information to the public may reduce TB and HIV related stigma
Kumar, 2009	Depressive/anxiety symptom (*OR: 0.72; 95% CI: 0.58-0.88; P=0.01*)<br><br>HIV regimen complexity index (*OR: 0.96; 95% CI: 0.93-1.0; P=0.05*)<br><br>Risky drink (*OR: 0.41; 95% CI: 0.22-0.76; P=0.01*)<br><br>Gender (female): (*OR: 0.59; 95% CI: 0.44-0.79; P=0.01*)<br><br>Age (18-34): (*OR: 0.79; 95% CI: 0.63-0.99; P=0.05*)<br><br>Restrictive ADLs (*OR: 0.48; 95% CI: 0.32-0.72; P=0.01*)	Antidepressants use (*OR: 1.09; 95% CI: 1.02-1.17; P=0.05*)<br><br>Age (>50) (*OR: 1.48; 95% CI: 1.05-2.10; P=0.05*)<br><br>White race (*OR: 0.1.77; 95% CI: 1.24-2.52; P=0.01*)<br><br>Social support (*OR: 1.04; 95% CI: 1.01-1.07; P=0.05*)	Number of HIV related diseases for which took medicine (*OR: 1.07; 95% CI: 0.97-1.19; P>0.15*)<br><br>Worked for pay 30 or more hours per week (*OR: 0.77; 95% CI: 0.58-1.01; P=0.1*)<br><br>Worked for pay less than 30 h per week (*OR: 0.77; 95% CI: 0.58-1.01; P=0.15*)
Walkup, 2007	Race (African Americans) (*OR=0.71; 95% CI=0.53-0.95; P≤0.05)*<br><br>Race (Latins) (*OR=0.61; 95% CI=0.43-0.85; P≤0.01)*	Antidepressant medication use (*OR=1.28; 95% CI=1.16-1.41; P≤0.01)*<br><br>Age (40-45) (*OR=1.49; 95% CI=1.01-2.21; P≤0.05)*<br><br>Age (⩾46) (*OR=1.97; 95%*	While the HAART adherence rate was low among patients with AIDS diagnosed with depression, prior month's antidepressant use increases odds of adherence.

		CI=1.22-3.17; P≤0.01)<br><br>Living in the high-prevalence area (*OR=1.80; 95% CI=1.37-2.37; P≤0.01)*<br><br>ACCAP membership (*OR=1.46; 95% CI=1.07-1.99; P≤0.05)*	
Cohn, 2002	**ART:**<br><br>Too busy (*OR=2.10; 95% CI=1.34-3.27; P<0.01*)<br><br>Gender (female) (*OR=1.65;95% CI=1.09-2.48；P<0.05*)<br><br>Age (younger) (*OR=1.03; 95% CI=1.01-1.06; P<0.01*)<br><br>Too many pills (*OR=1.62; 95% CI=1.12-2.36; P<0.05*)<br><br>Getting a particular treatment or drug (*OR=1.36; 95%CI=1.01-1.84; P<0.05*)<br><br>Not satisfied with friend/family support (*OR=1.50; 95% CI=1.02-2.22; P<0.05*)<br><br>Skip medication history (*OR=1.25; 95% CI=1.15-1.36; P<0.01*)<br><br>**MAC therapy:**<br><br>Gender (female) (*OR=1.64; 95% CI=1.11-2.41; P<0.05*)<br><br>Age (younger) (*OR=1.02; 95% CI=1.01-1.05; P<0.01*)<br><br>Skip medication history (*OR=1.09; 95% CI=1.01-1.18; P<0.01*)<br><br>Concerned medication side effects (*OR=1.51; 95%*<br><br>*CI=1.12-2.05; P<0.01*)<br><br>Recognize the outcomes of not taking medication (*OR=1.50; 95% CI=1.13-1.98; P<0.01*)<br><br>Cocaine use history (*OR=1.49; 95% CI=1.13-1.97; P<0.05*)<br><br>Often overwhelmed by problems (*OR=1.23; 95% CI=1.00-1.50; P<0.01*)		Nonadherence was more common among patients who received MAC prophylaxis than it was among those who received antiretroviral therapy. Self-reported nonadherence to antiretroviral therapy and MAC prophylaxis was significantly associated with the development of an AIDS-related complication or death.
Eldred, 1998		**ART:**<br><br>Taking medication ≤ twice a day (*OR=1.44;*	

		95% CI=1.01-1.96; P≤0.05)<br><br>Take medication not at home (*OR=1.41; 95% CI=1.04-2.00; P≤0.05*)<br><br>Belief in self ability to take medication consistently (*OR=1.57; 95% CI=1.13-2.17; P≤0.05)*<br><br>**PCP prophylaxis:**<br><br>Presence of family (*OR=2.39; 95% CI=1.01-5.63; P≤0.05*)<br><br>Belief in self ability to take medication consistently (*OR=2.87; 95% CI=1.44-1.78; P≤0.05)*	

Abbreviations:

ACCAP: AIDS Community Care Alternatives Program; ADAP: AIDS Drug Assistance Program; ADE: adverse drug effects; ADLs: activities of daily living; AI: active ingredients; AOR: adjusted odds ratio; ART: antiretroviral therapy; CESD: Centers for Epidemiological Studies Depression scale; CI: confidence interval; CPT: cotrimoxazole preventive therapy; HAART: highly active antiretroviral therapy; IPT: isoniazid preventive therapy; IVDU: intravenous drug use; MAC: mycobacterium avium complex; MRCI: medication regimen complexity index; MTR: multiple tablet regimen; M/XDR-TB: multi or extensively drug-resistant TB; NNRTI: non-nucleoside reverse transcriptase inhibitors; OR: odds ratio; P: P-value; PCP: pneumocystis carinii pneumonia; r: correlation coefficient; PLWH: people living with HIV; STR: single-tablet regimen; TB: tuberculosis

†: nonadherence *vs.* adherence
